# Knowledge, Attitudes and Perceptions of Medical Students on Antimicrobial Stewardship

**DOI:** 10.3390/antibiotics9110821

**Published:** 2020-11-17

**Authors:** Panagiotis Efthymiou, Despoina Gkentzi, Gabriel Dimitriou

**Affiliations:** Department of Paediatrics, Patras Medical School, University of Patras, 26504 Rio Achaia, Greece; panosefth@upatras.gr (P.E.); gdim@upatras.gr (G.D.)

**Keywords:** knowledge, attitudes, medical students, antimicrobial, stewardship, prescribing, antibiotics

## Abstract

Antimicrobial Resistance (AMR) is an ongoing threat to modern medicine throughout the world. The World Health Organisation has emphasized the importance of adequate and effective training of medical students in wise prescribing of antibiotics Furthermore, Antimicrobial Stewardship (AMS) has been recognized as a rapidly growing field in medicine that sets a goal of rational use of antibiotics in terms of dosing, duration of therapy and route of administration. We undertook the current review to systematically summarize and present the published data on the knowledge, attitudes and perceptions of medical students on AMS. We reviewed all studies published in English from 2007 to 2020. We found that although medical students recognize the problem of AMR, they lack basic knowledge regarding AMR. Incorporating novel and effective training methods on all aspects of AMS and AMR in the Medical Curricula worldwide is of paramount importance.

## 1. Introduction

Antimicrobial Resistance (AMR) is an ongoing threat to modern medicine throughout the world with a negative effect on patient treatment outcome. Pathogens are developing mechanisms of resistance, making it difficult to treat common infectious diseases like pneumonia, tuberculosis and foodborne diseases [1,2,3,4]. Antibiotic prescribing is determined by various factors, including the socio-cultural and socio-economic factors of each country and the beliefs of patients and professionals regarding antibiotic use [5,6]. In many developing countries, there is shortage of appropriate diagnostic tools, resulting in the unnecessary administration of antibiotics [7,8]. It has also been observed that the insufficient regulatory policies of each country can cause an increase in over-the-counter antibiotics [9]. The World Health Organisation (WHO) has clearly emphasized the importance of adequate and effective training of medical students in the wise prescribing of antibiotics [10]. In 2015, the WHO endorsed the Global Action Plan on Antimicrobial Resistance, which highlights the importance of training all healthcare professionals regarding AMS [11]. It is vital that healthcare students are aware of the challenges posed by AMR, and that there are investments in training them on topics relevant to responsible antibiotic use in their chosen specialties [11].

Thus, future medical professionals have to be prepared appropriately in order to face the challenges of antimicrobial use in everyday clinical practice [12]. Nowadays, medical education incorporates thorough knowledge of infectious diseases and diagnosis, as well as antibiotic utilization and pathogen resistance mechanisms. All the above-mentioned fields of knowledge are highly required for medical students [13]. Furthermore, Antimicrobial Stewardship (AMS) has been recognized as a rapidly growing field in medicine that sets a goal of rational use of antibiotics in terms of dosing, duration of therapy and route of administration [13,14,15].

Taking into consideration the importance of medical education on AMS and AMR, we undertook the current review to systematically summarize and present the published data on knowledge, attitudes and perceptions of medical students on AMS.

## 2. Results

In the present review, we included 25 studies. Fifteen of them were focused exclusively on medical students (Table 1), whereas ten were conducted on healthcare professional students with the inclusion of medical students amongst them (Table 2). All studies included final year medical students, three of them also included prefinal students, and three studies included medical students from all years of medical school. We found studies from all over the world in countries of Europe, America, Africa, Asia and Oceania. As for the studies focusing exclusively on medical students, five were conducted in Asia [16,17,18,19,20], three in Africa [21,22,23], one in Oceania [24], five in Europe [10,25,26,27,28] and finally one study in the USA [11]. All studies used questionnaires as research tools so they could collect data regarding the knowledge, attitudes, and perceptions of medical students about AMS. More precisely, questionnaires included questions on self-perceived preparedness to prescribe antibiotics and the importance of AMS. Knowledge regarding antibiotics was, in most cases, tested by clinical scenarios or general questions. The oldest study was conducted in 2012 and the latest in 2020. We also included 10 multidisciplinary studies (Table 2) that incorporated medical, pharmacy, dental, veterinary students, medical interns and physicians [29,30,31,32,33,34,35,36,37,38].

A study in a Japan University in Japan showed that 92.6% of their medical students had sufficient knowledge of the mechanism of actions of antibiotics. However, 30% of the respondents worryingly answered that antibiotics could be used to treat viral infections and around half of them believed that they could be administered for the common cold. We should point out that wrong answers were significantly lower among final year medical students compared to the first-year students. Lastly, only 6.5% of the students were aware of the AMR plan that was promoted by the Japanese government [18].

In 2015, a survey in eight universities from Australia showed that 70% of medical students felt more confident regarding their knowledge in cardiology compared to infectious diseases, where 54% were confident of their knowledge. As for clinical knowledge, students scored better in cardiovascular disease questions (64%) contrary to antibiotic prescribing questions (45%). In addition, nearly all students were aware of guidelines referring to antimicrobial prescribing. A negative aspect of this study was the small number of participants, which may not give an accurate perception of the country’s undergraduate students [24].

Our research revealed two studies from China, a country with the second largest antibiotic consumption in the world and high rates of dispensable use of antibiotics. The majority (92%) of medical students from Central China agree that misuse of antibiotic treatment increases AMR, where 67% found their training useful in antimicrobial management. Once again, the percentage of correct answers in a broad spectrum of questions around the treatment of different infections was low, with the total correct percentage being 34% [16]. Another study from China showed that 27% of medical students reported self-medication with antibiotics. Additionally, 64% of the respondents reported that they were stocking antibiotics for personal use, whilst 97% have bought antibiotics without medical prescription in the past [17].

The studies that we found from Europe were conducted under the European Society of Clinical Microbiology and Infectious Diseases Study Group of Antimicrobial Policies (ESGAP/ESCMID). In 2012, seven European medical schools competed an online survey which reported that medical students felt more confident about infection diagnosis in comparison to treatment, e.g., administration route, duration, necessity of antimicrobial use. A high percentage of them (83%) believed that incidence of methicillin-resistant *Staphylococcus aureus* (MRSA) bacteraemias has increased in their country, which was not true. Moreover, medical students overestimated possible death rate resulting from resistant bacteria compared to lung cancer. Nearly all medical students (98%) believed that AMR would be a major issue in the foreseeable future [10]. In another study, we have more detailed results from France, where 64% of the respondents were aware of the implementation of antibiotic guidelines in their hospital and 62% have used them in practice. Furthermore, 94% believed that AMR is a national problem. It should be noted at this point that this study was limited due to the small number of participants [27]. A Spanish study showed that 40.4% of medical students claimed that they would prescribe an antibiotic without consulting guidelines. On the other hand, only 24.3% believed they had adequate training regarding rational use of antibiotics [26]. In 2015, 29 European countries participated in an online survey of ESGAP to assess the preparedness of medical students using antibiotics in a judicious way. Countries that incorporate guidelines in clinical practice, like the United Kingdom, reported a higher rate of preparedness. This conclusion can also be related to quality of education on the use of antibiotics. Hence, there was some variability between different European countries [25]. For example, a study comparing France and Sweden showed that French medical students were less likely to feel prepared compared to the Swedish students and they suggest a need for more focused education in the field. A hypothesis that originated from this study claims that this concept may come from the fact that Swedish medical students tend to have more clinical exposure to the approach of antibiotic management [28]. In general, 37.3% of medical students requested more education on this subject. This is one of the greatest studies considering the preparedness of medical students on a topic [25].

Another study from India revealed that medical students have insufficient knowledge about AMR and AMS (39.7% of prefinal and 54.8 of final year medical students answered correct). Most of the students knew that viral infections cannot be treated with antibiotics. However, many of them had did not know how to treat and prevent MRSA infections effectively and could not recall the acronym *Enterococcus faecium*, *Staphylococcus aureus*, *Klebsiella pneumoniae*, *Acinetobacter baumannii*, *Pseudomonas aeruginosa*, and *Enterobacter* species (ESKAPE) pathogens [19]. Additionally, a study from a medical school in Nigeria showed that 64.7% of the students had a good understanding of antibiotic resistance and use, although a mere 39% of them would treat common cold with antibiotics. Furthermore, only 8.2% of the students took medical consultation before taking antibiotics [21]. In a multi-centre, cross-sectional study in Egypt, 43% of the respondents considered that skipping doses of antibiotic treatment does not affect AMR, which is a common misconception. Around 40% of the students would use antimicrobials for a sore throat. Furthermore, students in final years performed better in the knowledge section of the questionnaire [23].

On a survey taking place in the USA in 2012, 90% of medical students from 3 universities (University of Miami, John Hopkins University, University of Washington) reported that they would like more education on antibiotics. More specifically, mean correct knowledge score was 51% which has been significantly affected by study sources that students used as learning methods. For instance, medical students scored high on questions regarding the use of antibiotics and management of community-acquired pneumonia. In contrast, they scored low in urinary tract infections and in questions for recognition of Clostridium difficile infection. Students who referred to physicians, pharmacists and those who used guidelines had better scores. Except from that only 15% of the students had followed a rotation in Infectious Disease department during their studies [12]. In addition, in a study from three medical schools in Thailand, 90% of students affirmed that the misuse of antibiotics is a major problem in their hospital and their country. The majority (98%) believed that they were capable of prescribing antibiotics, while 71.4% would feel stressed when prescribing. Over 10% of medical students reported that they have never been taught the principles of prudent use of antibiotics and AMR [20].

Last but not least, a study including three medical Schools in Africa showed that one out of three medical students did not feel confident enough on antibiotic prescribing [22]. On the other hand, students claimed that antibiotic overuse (63%) and resistance (61%) is an essential problem in their hospital, while 92% believed that antibiotics are overused and that the AMR is a crucial issue in the South African region. They also had the perception that hand hygiene is not a large contributing factor to AMR [22].

## 3. Discussion

In the current review, our main goal was to reveal if and how medical students were taught the basic principles of AMS. Nowadays, we are living in an era of excessive and often irrational usage of antibiotics in some settings, resulting in a significant increase in AMR. As this is an intercontinental issue, several studies conducted around the world give us valuable data for the preparation of future medical professionals. Our research has shown noteworthy findings regarding the knowledge, attitudes and perception of medical students.

### 3.1. Knowledge

It is clearly shown that medical students today lack adequate basic and clinical knowledge on the principal concepts of infectious diseases. In all parts of the world, medical students score relatively low in the relevant knowledge questionnaires as well as in the included clinical scenarios, making it clear that there is a room for improvement in medical education in both developed and developing countries [12,20,23,33]. Notwithstanding that medical students know that inappropriate use of antibiotics increases bacterial resistance, there are some common misperceptions [22]. For example, many of them claim that antibiotics can be used for treating viral infections and common flu [23,33]. It is widely known that those practices can increase inappropriate antimicrobial usage, resulting in high rates of AMR. In contrast to the above, diagnosis seems to be an easier task for medical students [10]. These knowledge gaps can occur due to the ineffective curriculums of each Medical School, which do not thoroughly cover the fundamentals of antibiotic usage, management, and duration of treatment, although the situation varies among different countries [39]. However, students who were in their final years of their studies in Medicine or had completed a clinical rotation in the Infectious Diseases Department of their hospital scored better in the knowledge section [16,18,33]. On the other hand, students who followed guidelines and reliable sources for learning tend to have more structured knowledge [34]. This finding implicates the importance of guidelines in undergraduate medical education in the early stage of the curricula. In any case, in all studies, medical students would appreciate more education in both basic science as well as clinical grounds so that they feel better prepared in their future tasks of everyday clinical practice [10,12,16,18,19,22,31,35,36,39]. It is of interest to note that, overall, following the WHO Global Action Plan on Antimicrobial Resistance [11], medical education about AMS might be essentially different in individual countries. Although we observed a trend of better knowledge in the field from 2015 onwards, we cannot accurately assess this difference as we have no country-specific data on educational changes and their potential impact. In addition, none of the included studies were designed to address this particular question, ideally comparing knowledge and attitudes on AMR with the same research tool and in the same setting before and after the WHO Global Action Plan on AMR.

### 3.2. Attitudes and Perceptions

Medical students do believe that rational use of antimicrobials is an essential aspect of their career to avoid the spread of AMR among pathogens [10,12,19,20,31]. These findings indicate that medical students and tomorrow’s doctors have a positive moral attitude towards this issue. They also believe that antibiotics should not be sold and administered without a medical prescription [17,19]. The recognition of AMR’s severity is the major factor that will guide and assist professionals in searching for efficacious ways of fighting mild as well as more severe infectious diseases. Although most students assess that antimicrobial overuse and resistance are worldwide issues, they tend to underestimate that this problem also exists in their hospital environment [12,16,40]. Such behaviors can lead to improper prescribing. Another interesting point is that a large percentage of medical students lack confidence and preparedness referred to antibiotic prescription [20,34,35]. This can correlate with each country’s AMR status, where students in countries with low resistance rates tend to feel more prepared, possibly because they are less exposed to severe infectious clinical challenges [36]. On the other hand, evidence exists that overconfidence in the field had a negative impact on antibiotic prescription [22,35].

### 3.3. Limitations of the Study

To the best of our knowledge, this is the first review that summarizes the relevant studies worldwide on AMS and medical students. However, our review has some limitations regarding data evaluation. First of all, medical students in the reviewed studies were questioned around different aspects of AMS, making it difficult to proceed to an equal assessment of the results. In addition to that, no international validated questionnaire exists in the field, which would obviously make the results of the studies more comparable and could be used for future studies. Moreover, different antibiotic policies and guidelines are in place in each country, and hence different behaviors and attitudes can be described. We should also take into consideration that learning and training resources do vary around the globe due to social and economic conditions. This can obviously have an impact on medical education strategies on AMS. Finally, depending on countries, the status of AMR is different, which may be related to medical education, but we do not have the data to proceed to such a comparison between countries.

### 3.4. Implications for Future Approach

With all above-mentioned issues, we understand the need to enhance medical education towards AMS. The majority of students worldwide consider traditional lectures and passive learning tools ineffective methods or an unsuccessful way of promoting knowledge on antibiotics and AMS [11,21,36,41]. Therefore, a more practical approach such as discussion of clinical scenarios and presence in clinical practice (e.g., clinical placements, clinical rotations [12,16,38]) seems to have a positive effect on knowledge of and attitude toward antibiotic usage and administration [20,35]. In addition, a more detailed teaching of basic microbiology knowledge could reinforce medical students with important information, which is necessary for a better understanding of antibiotics [42]. Another useful intervention would be cooperation between medical universities in order to exchange educational approaches, and also between medical schools and faculties like pharmacy schools (e.g., interprofessional workshops and simulations between medical and pharmacy students), so they can learn more about the uses and specific features of antibiotics, by introducing principles and the importance of AMR [43,44,45]. As far as time organization is concerned, curricula studies have shown that early introduction of AMR teaching as well as repetitive and enriched training upon antibiotic resistance, diagnosis, management, prescribing and communication skills would lead to a more comprehensive understanding of the challenges and complexities of infectious diseases [43,46,47,48]. Moreover, e-learning and online education about AMS is a desirable and effective method according to medical professionals and students [41,49], although a European survey questions the successfulness of this means [35,50]. Furthermore, a study based on a seminar for medical students included real patients and their advocates. Post this seminar, students believed that hearing patients’ stories is an effective way of learning more about AMR and the importance of stewardship [51]. There should also be a change regarding the learning tools and resources which medical students study during their years of medical school. Of note, students who follow the updated guidelines [52] and those who referred to medical and pharmacy specialists tended to have a more completed and updated knowledge on AMS [12]. Another helpful implication would be to encourage medical students to get involved in undergraduate research to acquire new academic skills and be aware of both AMR and AMS [53]. However, there is need for further future assessment of current medical training methods so we can make clear assumptions about their effectiveness [54].

## 4. Materials and Methods

We reviewed all studies published in English from 2007 to 2020. The studies were included if they contained original results or had exceptional content with particular emphasis on studies Knowledge, Attitude, Perceptions (KAP) studies.

The initial search was conducted in the PUBMED and Scopus databases and the last search was performed on 1 September 2020. The following key words and their combinations were used for the search: antimicrobial stewardship*, medical students*, knowledge*, attitudes*, perceptions*. Duplicate publications were identified and removed.

**Figure 1:**
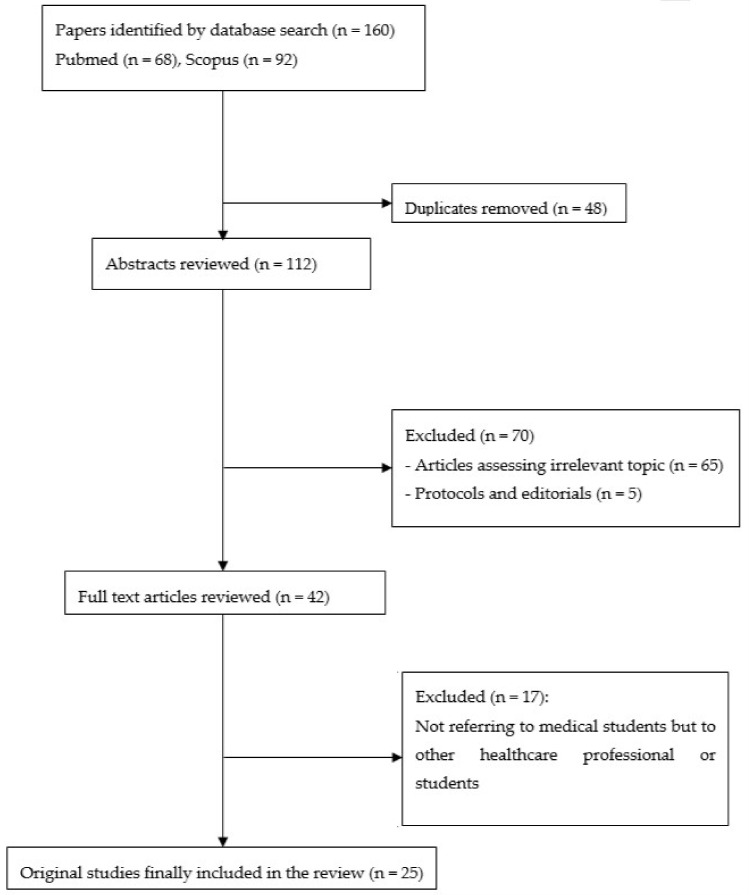
Flowchart of methodology and included studies.

We identified 160 potentially relevant articles through database searches. Of these, there were 48 duplicates and 70 were excluded on the basis of title and abstract screening. We also excluded studies that investigated training methods for promoting AMS. Hence, only studies with quantitative results regarding the knowledge and attitudes of medical students regarding AMS were included. We focused on medical students and no other health-related undergraduate students (e.g., pharmacy or dental students). Studies on the knowledge and attitudes of other healthcare professionals or students (i.e., non-medical students) are listed as Supplementary Materials (Table S1). Moreover, protocols and editorials were excluded. Figure 1 describes the details of the methodology and excluded studies.

## 5. Conclusions

Education on AMS is an emerging fundamental value for medicine around the world due to rapidly increasing AMR. Today’s medical professionals will hand over the baton to medical students and hope for a greater improvement in AMR and antibiotic usage. Although medical students recognize the imminent issue of excessive resistance, they also lack basic knowledge regarding AMR. Consequently, at this time we should provide knowledge and confidence to medical students so that they will be able to face ongoing daily clinical challenges in the future. This could be achieved by incorporating novel and effective training methods on all aspects of AMS and AMR in the medical curricula worldwide.

## Figures and Tables

**Table 1:** Studies assessing knowledge, attitudes and perceptions of medical students regarding Antimicrobial Stewardship (AMS).

Country/Study Years	Type of Study	Number of Participants	Main Findings/Outcomes	Reference
France (2012)	Cross-sectional	54 medical students (prefinal and final year)	Medical students feel more confident diagnosing infections than treating them. Four in five wanted more education on antimicrobial use. Almost all of them (96%) found unnecessary prescription unethical. The majority think that AMR is a national problem.	Dyar et al. [27]
USA (2012)	Cross-sectional	317, 4th year medical students at University of Miami, John Hopkins University and the University of Washington	The majority perceive that antimicrobial use is very important and would like more education on it. Around half of them answered correctly in knowledge-related questions depending on their educational sources. Those who completed an elective in infectious diseases felt more confident, however, this had no significant connection with better knowledge scores.	Abbo et al. [12]
Europe (2012)	Multi-centre, cross-sectional study	338 final year medical students from 7 European universities	Students wanted more education on antibiotic prescribing. Most of the students incorrectly believed that MRSA bacteraemia had increased in their country the last decade. They overestimated the burden caused by resistant bacteria compared to lung cancer.	Dyar et al. [10]
China (2015)	Multi-centre, cross-sectional	611, 4th year medical students from 5 teaching university hospitals of Central China	Medical students wish for more training on antimicrobial use through AMS programs and clinical rotations. No significant difference in knowledge of antimicrobial use between students who used textbooks and smartphones as teaching material.	Yang et al. [16]
France, Sweden (2015)	Multi-centre, cross-sectional	2085 medical students in France and 302 students in Sweden (all in final year)	Preparedness for antimicrobial use was higher in Sweden than in France. Students in France perceive that they need further education on antibiotic use. Swedish students had more working experience at the hospital than French students.	Dyar et al. [28]
Europe, 29 countries (2015)	Multi-centre, Cross-sectional	7328 final year medical students from 179 medical schools	Medical students tend to be more confident in diagnosis of infections than choosing an accurate therapeutic plan. Over a third of the students wanted more education on antimicrobial use. Northern countries show a higher amount of confidence. Clinical cases and small-group teaching sessions are preferred by students.	Dyar et al. [25]
China (2015)	Multi-centre, Cross-sectional	1819 medical students in 6 Universities (all grades of medical school)	The majority of students self-medicated their own illnesses and had a stock of antibiotics, in most cases without medical prescription. One in seven used antibiotics for common cold and asked their doctors for antibiotics.	Hu et al. [17]
Australia (2015)	Multi-centre, cross-sectional	163 final-year medical students from 8 universities in Australia	54% of medical students were ‘confident’ in their knowledge of infectious disease, while 70% were confident in Cardiology. They believe hands on clinical practise more helpful than lectures. Most of them were aware of AMR.	Weier et al. [24]
Thailand (2016)	Multi-centre, cross-sectional	455 final year medical students in 3 medical schools	Almost all of the participants perceive that prescribing board-spectrum antibiotics increases AMR. Half of them answered correct questions related to antibiotic use. Students evaluate bedside teaching as the most effective learning method for appropriate usage of antibiotics.	Chuenchom et al. [20]
South Africa (2017)	Multi-centre, cross-sectional	289 final year medical students in 3 medical schools	The majority assert that antibiotics are overused and that AMR is a major problem in South Africa. Only a third of the participants feel confident in prescribing antibiotics, which is related to use og guidelines and contact with infectious diseases specialists. Medical students who followed prescribing guidelines had a better score in the knowledge section of the questionnaire.	Wasserman et al. [22]
Nigeria (2018)	Cross-sectional	184 medical students (prefinal, final year) at Ebonyi State University, Nigeria	The vast majority wanted more training on antibiotic usage and resistance. Almost half of them had practical training on antibiotic use. Around 2/3 had adequate knowledge on antibiotic use and resistance. However, a high level of false antibiotic management was reported.	Oketo-Alex et al. [21]
Egypt (2018)	Multi-centre, Cross-sectional	963 medical students (all years) from 25 medical schools of Egypt	Almost all students had sufficient knowledge and attitudes towards AMR. Around 40% believed that antibiotics can treat the common cold. Nearly half of them would stop antibiotics when starting to feel well.	Assar et al. [23]
Spain (2019)	Multi-centre, cross-sectional	441 final year medical students from 21 Spanish medical schools	Medical students feel more confident diagnosing than treating infectious diseases. They want additional education regarding antibiotic usage.	Sánchez-Fabra et al. [26]
Japan (2019–2020)	Cross-sectional	661 undergraduate medical students at Okayama University Medical School (1st to 6th year)	90% of medical students knew the mechanism of action of antibiotics. 30% of all medical students believed that antibiotics could treat viral infections, while 46.4% considered antibiotics as treatment for the common cold. AMR plan awareness was poor (6.5%).	Hagiya et al. [18]
India (2020)	Cross-sectional	197 medical students (93 prefinal year and 104 final year) of a teaching institute in Southern Indian city	Insufficient knowledge of medical students about AMR and AMS. However, they recognize that appropriate use of antibiotics is essential, as well as training on AMS.	Meher et al. [19]

**Table 2:** Studies assessing knowledge, attitudes and perceptions of medical students as well as other healthcare professional students regarding AMS.

Country/Study Years	Type of Study	Number of Participants	Main Findings/Outcomes	Reference
India (2015)	Multidisciplinary, cross-sectional	120 medical and 48 dental students	98% of medical students had positive attitude and knowledge towards AMS.	Sharma et al. [34]
India (2017)	Multidisciplinary, cross-sectional	198 medical students, 89 dental students, 89 nursing students, 80 pharmacy students	A great amount of healthcare students tend to use antibiotics over the counter. 25% of medical students reported self-medication. 33% of medical students did not finish the course of antibiotics.	Virmani et al. [30]
UK (2018)	Multidisciplinary, multicentre, cross-sectional	255 students, 165 pharmacy students, 71 veterinary students, 12 medical students, 11 dental students, 3 physicians, 2 nurses	95% of the students believe that AMR will be a major issue in their future clinical practice. One out of five students felt confident about their knowledge about antimicrobial use	Dyar et al. [31]
Croatia (2018)	Multidisciplinary, cross-sectional	115 medical students 46 pharmacy students	90.7% of students believed that antibiotics are overused. No difference in average knowledge score between medical and pharmacy students.	Rusic et al. [32]
USA (2018)	Single-centre, cross-sectional	103 participants (31 medical students, 57 medical residents, 12 attending physicians)	85% of the responders believed that it would be beneficial for medical staff to be trained further and 75% agreed on the need for AMS education. Medical students had the lowest mean of correct answers compared to medical interns and physicians	Beatthy et al. [36]
USA (2019)	Single-centre, cross-sectional	50 medical students, 30 medical residents, 6 nurse practitioners	Lack of communication among senior and junior practitioners was observed regarding decision of antimicrobial treatment.	Smoke et al. [29]
Pakistan (2019)	Multidisciplinary, cross-sectional	247 prefinal and final year medical students, 203 prefinal and final year pharmacy students	79% of the participants believed that AMS is an important issue in their hospital. Knowledge of antimicrobial use and resistance was higher in pharmacy students than medical students. More pharmacy students would like further training regarding AMS compared to medical students.	Saleem et al. [37]
United Arab Emirates (2019)	Multidisciplinary, cross-sectional	Medical students, pharmacy students, veterinary students, dental students, engineering, technology and law students	Knowledge, attitude and perception score was better among medical students (58%) than in other groups of students (52%). High rates of antibiotic self-medication (38,2%).	Jairoun et al. [38]
Iran (2020)	Multicentre, cross-sectional	126 responders including infectious diseases practitioners, surgeons, medical interns, medical students, general practitioners, microbiology lab technicians and PhD students.	88.1% of participants agreed on establishment of local guidelines. 94.4% claimed that training regarding proper antibiotic use can bring positive effects on reducing AMR.	Firouzabadi et al. [35]
Rwanda (2020)	Multidisciplinary, cross-sectional	115 medical students, 41 dental students.	83% did not have knowledge of AMS. 23% did not agree that excessive antibiotic use can lead to AMR. 50% claimed that antibiotics can be used for pain and inflammation.	Nisabwe et al. [33]

## Supplementary Materials

The following are available online at https://www.mdpi.com/2079-6382/9/11/821/s1, Table S1: Studies on knowledge and attitudes of other healthcare professionals and non-medical students.
