# Supplementary material for: Knowledge, Attitudes and Perceptions of Medical Students on Antimicrobial Stewardship
Source: Antibiotics (Basel). 2020 Nov 17;9(11):821. doi: 10.3390/antibiotics9110821 (PMC7698472; doi:10.3390/antibiotics9110821)
Supplement: Supplementary file 1 [file antibiotics-09-00821-s001.pdf]

Supplementary Material

Table S1. Studies on knowledge and attitudes of other healthcare professionals and non-medical students

Author-Year	Study Summary	Reason for Exclusion
Childs-Kean et al. 2018 [1]	Review of the published evidence describing and medical and pharmacy trainees' involvement in AMS and call for future research in this area.	The study included medical interns, fellows and pharmacy residents.
Wilcock et al. 2019 [2]	The role of nurses and midwives and their contribution to AMS.	The study included nurses and midwives.
Kufel et al. 2018 [3]	Multicenter-cross-sectional study assessing the different AS program in USA for pharmacy students. Interprofessional collaboration may be needed.	The study included AMS programs for pharmacy students
Bowes et al. 2014 [4]	Single-Center study. AMS programs should be included in the everyday clinical practice of pediatricians.	The study included physicians and medical interns
Revolisnki et al. 2020 [5]	Understanding of AS by pharmacy students and how this can change after exposure to AMS techniques.	The study included pharmacy students.
Mersha et al. 2018 [6]	Multi center, cross-sectional study. Medical interns want more education regarding AMS.	The study included medical interns
Chahine et al. 2015 [7]	Review of the published literature to propose a model to engage pharmacy students, residents and fellows in AMS	The study included pharmacy students, residents and fellows.
Charani et al.2019 [8]	International survey. AS policies are found more often in hospitals rather in primary care. Post graduate training is needed in all disciplinaries.	The study included doctors, nurses, pharmacists and others (researchers, students and members of the public). No clear data for medical students.
Smith et al. 2019 [9]	Knowledge and perceptions of veterinary students about AMR. The gap between theoretical knowledge and practical scenarios should be diminished.	The study included veterinary students.
Justo et al. 2014 [10]	Knowledge and attitudes of pharmacy students regarding appropriate use of antibiotics	The study included pharmacy students
Inácio et al. 2017 [11]	Master of Pharmacy students' knowledge and awareness of antibiotic use, resistance and stewardship	The study included master pharmacy students
Rábano-Blanco et al. 2019 [12]	Nursing students' knowledge and awareness of antibiotic use, resistance and stewardship	The study included nursing students.
Bonsignore et al. 2018 [13]	Perspectives for anesthesiologists in hospital hygiene	The study included anesthesiology specialists.
Siegfried et al. 2017 [14]	Role of postgraduate year 2 pharmacy residents in providing weekend antimicrobial stewardship coverage in an academic medical center	The study included pharmacy students.
Khan et al. 2017 [15]	Undergraduate antimicrobial stewardship training for pharmacy students: Creating a foundation for	The study included undergraduate pharmacy students.

	containment of antimicrobial resistance in South Africa	
Almulhim et al. 2019 [16]	Optimization of antibiotic selection in the emergency department for urine culture follow ups, a retrospective pre-post intervention study: Clinical pharmacist efforts	The study included pharmacists' strategies.
Peel et al. 2020 [17]	Perioperative antimicrobial decision making: Focused ethnography study in orthopedic and cardiothoracic surgeries in an Australian hospital	The study did not include medical students

References:

1. Childs-Kean, L.M.; Briggs, H.L.; Cho, J.C. All aboard!: Involvement of medical and pharmacy trainees in antimicrobial stewardship. *Infect Control Hosp Epidemiol*. **2018**, 40, 200-205, DOI: 10.1017/ice.2018.332
2. Wilcock, M.; Powell, N.; Underwood, F. Antimicrobial stewardship and the hospital nurse and midwife: How do they perceive their role? *Eur J Hosp Pharm*. **2019**, 26, 89-92, DOI: 10.1136/ejhpharm-2017-001312
3. Kufel, W.D.; Jeffres, M.N.; MacDougall, C.; Cho, J.C.; Marx, A.H.; Williams, D.M. Antimicrobial stewardship education in US colleges and schools of pharmacy. *J. Antimicrob. Chemother*. **2018**, 73, 2252-2258, DOI: 10.1093/jac/dky166
4. Bowes, J.; Yasseen A.S.; Barrowman, N.; Murchison, B.; Dennis, J.; Moreau, K.A.; Varughese, N.; Le Saux, N. Antimicrobial stewardship in pediatrics: Focusing on the challenges clinicians face. *BMC Pediatr*. **2014**, 14:212, DOI: 10.1186/1471-2431-14-212
5. Revolinski, S.; Pawlak, J.; Beckers, C. Assessing Pharmacy Students' and Preceptors' Understanding of and Exposure to Antimicrobial Stewardship Practices on Introductory Pharmacy Practice Experiences. *Pharmacy (Basel).* **2020**, 8, DOI: 10.3390/pharmacy8030149
6. Mersha, A.G. Attitude and perception of medical interns about antimicrobial resistance: A multi center cross-sectional study in Ethiopia 11 Medical and Health Sciences 1117 Public Health and Health Services. *Antimicrob. Resist. Infect. Control*. **2018**, 7, DOI: 10.1186/s13756-018-0443-9
7. Chahine, E.B.; El-Lababidi, R.M.; Sourial, M. Engaging pharmacy students, residents, and fellows in antimicrobial stewardship. *J. Pharm. Pract*. **2015**, 28, 585-591, DOI: 10.1177/0897190013516506
8. Charani, E.; Castro- Sanchéz, E.; Bradley, S.; Nathwani, D.; Holmes, A.H.; Davey, P. Implementation of antibiotic stewardship in different settings - results of an international survey. *Antimicrob Resist Infect Control.* **2019**, 8, DOI: 10.1186/s13756-019-0493-7
9. Smith, P.W.; Agbaje, M.; LeRoux-Pullen, L.; van Dyk, D.; Debusho, L.K.; Shittu, A.; Sirdar, M.M.; Fasanmi, O.G.; Adebowale, O.; Fasina, F.O. Implication of the knowledge and perceptions of veterinary students of antimicrobial resistance for future prescription of antimicrobials in Animal health, South Africa. *J S Afr Vet Assoc.* **2019**, 90, DOI: 10.4102/jsava.v90i0.1765
10. Justo, J.A.; Gauthier, T.P.; Scheetz, M.H.; Chahine, E.B.; Bookstaver, E.B.; Gallagher, J.C.; Hermsen, E.D.; DePestel, D.D.; Ernst, E.J.; Jacobs, D.M.; Esterly, J.S.; Suda, K.J.; Olsen, K.M.; Abbo, L.M.; MacDougall, C. Knowledge and attitudes of doctor of pharmacy students regarding the appropriate use of antimicrobials. *Clin. Infect. Dis*. **2014**, 59, 162-169, DOI: 10.1093/cid/ciu537
11. Inácio. J.; Barnes, L-M.; Jeffs, S.; Castanheira, P.; Wiseman, M.; Inácio. S.; Bowler, L.; Lansley, A. Master of Pharmacy students' knowledge and awareness of antibiotic use, resistance and stewardship. *Curr. Pharm. Tech. Learn.* **2017**, 9, 551-559, DOI: 10.1016/j.cptl.2017.03.021

12. Rábano-Blanco, A.; Domínguez-Martís. E.M.; Mosteiro-Miguéns. D.G.; Freire-Garabal, M.; Novío, S. Nursing students' knowledge and awareness of antibiotic use, resistance and stewardship: A descriptive cross-sectional study. *Antibiotics*. **2019**, 8, DOI: 10.3390/antibiotics8040203
13. Bonsignore, M.; Alefelder, .C.; Pausner, N.; Gastmeier, P.; Nachtigall, I. Perspectives for anesthesiologists in hospital hygiene. *Anaesthesist*. **2018**, 67, 758-765, DOI:10.1007/s00101-018-0478-6
14. Siegfried, J.; Merchan, C.; Scipione, M.R.; Papadopoulos, J.; Dabestani, A.; Dubrovskaya, Y. Role of postgraduate year 2 pharmacy residents in providing weekend antimicrobial stewardship coverage in an academic medical center. *Am. J. Health-Syst. Pharm*. **2017**, 74, 417-423, DOI: 10.2146/ajhp160133
15. Khan, Y.; Boschmans, S.A.; McCartney, J.; Coetzee, R. Undergraduate antimicrobial stewardship training for pharmacy students: Creating a foundation for containment of antimicrobial resistance in South Africa. *S. Afr. Med. J.* **2017**, 107, 722, DOI: 10.7196/SAMJ.2017.v107i9.12649
16. Almulhim, A.S.; Aldayyen, A.; Yenina, K.; Chiappini, A.; Khan, T.M. Optimization of antibiotic selection in the emergency department for urine culture follow ups, a retrospective pre-post intervention study: Clinical pharmacist efforts. *J. Pharm. Policy Pract*. **2019**, 12, DOI: 10.1186/s40545-019-0168-z
17. Peel, T.; Watson, E.; Cairns, K.; Lam, H.Y.A.; Li, H.Z.; Ravindran, G.; Seneviratne, J.; Daly, D.; Liew, S.; McGiffin, D.; Myles, P.; Ayton, D. Perioperative antimicrobial decision making: Focused ethnography study in orthopedic and cardiothoracic surgeries in an Australian hospital. *Infect. Control. Hosp. Epidemiol*. **2020**, 41, 645-652, DOI: 10.1017/ice.2020.48
